# SOCS3 inhibits the pathological effects of IL-22 in non-melanoma skin tumor-derived keratinocytes

**DOI:** 10.18632/oncotarget.15629

**Published:** 2017-02-22

**Authors:** Stefania Madonna, Claudia Scarponi, Martina Morelli, Rosanna Sestito, Pasqualina Liana Scognamiglio, Daniela Marasco, Cristina Albanesi

**Affiliations:** ^1^ Laboratory of Experimental Immunology, IDI-IRCCS, Fondazione “Luigi M. Monti” (FLMM), Rome, Italy; ^2^ Department of Pharmacy, CIRPEB, University of Naples “Federico II”, Naples, Italy; ^3^ Current address: Preclinical Models and New Therapeutic Agents Unit, Regina Elena National Cancer Institute, Rome, Italy

**Keywords:** SOCS1, SOCS3, non-melanoma skin cancers, IL-22 signaling, healthy and transformed keratinocytes

## Abstract

Basal cell carcinomas (BCC) and squamous-cell carcinomas (SCC) are common malignancies in humans, caused by neoplastic transformation of keratinocytes of the basal or suprabasal layers of epidermis, respectively. Tumor-infiltrating lymphocytes (TILs) are frequently found in BCC and SCC, and functionally promote epithelial carcinogenesis. TILs secreting IL-22, in particular, participate to BCC and SCC growth by inducing keratinocyte proliferation and migration, as well as the expression of inflammatory, anti-apoptotic and pro-angiogenic genes.

In this study, we identified SOCS3 as a valid candidate to be manipulated for suppressing tumorigenic functions in BCC and SCC. We found that SOCS3 and SOCS1 expression was reduced *in vivo*, in tumor lesions of BCC and SCC, as compared to other skin inflammatory conditions such as psoriasis, despite the high number of IL-22-secreting TILs. Moreover, IL-22 was not able to induce *in vitro* the transcriptional expression of SOCS3 in BCC-or SCC-derived keratinocytes, contrarily to healthy cells. Aimed at rescuing SOCS3 activity in these tumor contexts, a SOCS3-derived peptide, named KIR-ESS, was synthesized, and its ability in suppressing IL-22-induced responses was evaluated in healthy and transformed keratinocytes. We found that KIR-ESS peptide efficiently suppressed the IL-22 molecular signaling in keratinocytes, by acting on STAT3 and Erk1/2 cascade, as well as on the expression of STAT3-dependent downstream genes. Interestingly, after treatment with peptide, both healthy and transformed keratinocytes could no longer aberrantly proliferate and migrate in response to IL-22. Finally, treatment of athymic nude mice bearing SCC xenografts with KIR-ESS peptide concomitantly reduced tumor growth and activated STAT3 levels. As a whole, these data provides the rationale for the use in BCC and SCC skin tumors of SOCS3 mimetics, being able to inhibit the deleterious effects of IL-22 in these contexts.

## INTRODUCTION

IL-22 belongs to a family of cytokines structurally related to IL-10, and it is secreted exclusively by immune cells, especially type-22 and type-17 T lymphocytes [1, 2]. IL-22 signals via a heterodimeric transmembrane receptor complex, consisting of IL-22R1 and IL-10R2 subunits and associated Janus kinase/signal transducers and activators of transcription (Jak/STAT) molecules, including Jak1, Tyk2, and STAT3 [3, 4]. In parallel, IL-22 can activate nuclear factor kappa B (NF-κB), mitogen-activated protein kinase (MAPK) and phosphatidylinositide 3-kinase-Akt mammalian target of rapamycin pathways [5, 6].

In the skin, IL-22 receptor complex is selectively expressed on keratinocytes [4], and following IL-22 binding, mediates the expression of genes principally downstream of STAT3, controlling keratinocyte proliferation, differentiation and apoptosis, as well as inflammatory responses [4, 7, 8]. IL-22R/IL-22 axis contributes to several pathophysiological processes in the skin, with functions depending on the disease context. For instance, in psoriasis, a chronic inflammatory skin disease, IL-22 is responsible for the marked hyperplasia and altered differentiation characterizing lesional epidermis [9]. IL-22 signalling also impacts on non-melanoma skin carcinomas (NMSC), including squamous cell carcinomas (SCC) and basal cell carcinomas (BCC) [10, 11]. In these tumour contexts, IL-22, locally released together with IL-17 by tumor-infiltrating lymphocytes (TILs), promotes epithelial carcinogenesis, since it activates proliferation and migration of transformed keratinocytes, as well as the expression of inflammatory and anti-apoptotic molecules which contribute to tumor progression [10, 12]. At the molecular level, IL-22-dependent effects in BCC- or SCC-derived keratinocytes are mainly mediated by STAT3, by extracellular signal-regulated kinases (Erk)1/2 and the anti-apoptotic Akt pathways [10].

It is known that full-activation of STAT3 requires its phosphorylation in both Tyr705 and Ser727 residues, and can be negatively regulated by the suppressor of cytokine signalling (SOCS)3, an endogenous modulator of intracellular Jak/STAT cascade broadly induced by cytokines [4, 13, 14]. SOCS3 belongs to a family of eight intracellular proteins, SOCS1-7 and cytokine-inducible SH2-containing protein (CIS), which share structural similarities and are characterized by a central SH2 domain, and by a unique motif in their carboxy-terminal module, the SOCS box, involved in the degradation of target proteins [15, 16]. Among SOCS members, only SOCS3 and SOCS1 contain a kinase inhibitory region (KIR), which inhibits kinase activity of specific Jaks by acting as a pseudosubstrate [17]. An extended SH2 subdomain, called ESS, within SOCS1 and SOCS3 also contributes to their interaction with Jaks [18].

In the last years, SOCS proteins have been identified as novel therapeutic targets to treat diseases that are driven by excessive cytokine responses, and are suspected to function as oncosuppressors, being silenced in many tumors [19, 20]. Small molecules that mimic the physiological action of the KIR domain of SOCS1 have been designed, and were shown to ameliorate inflammatory processes by down-regulating specific immune and pro-tumor responses both *in vivo* and *in vitro* [21, 22]. SOCS1-KIR and its analogues also show promise in certain skin diseases characterized by local aberrant proliferation. For instance, SOCS1 mimetic peptides suppress inflammatory responses triggered by cytokines in epidermal keratinocytes of psoriatic skin [23, 24]. Accordingly, our group recently developed a SOCS1-KIR mimetic, named PS-5, that significantly blocked IFN-γ-induced Jak2 and STAT1α activation, as well as expression of immunomodulatory molecules and chemokines in *in vitro* and *ex-vivo* experimental models of psoriasis [23, 24].

Although SOCS inhibitory action on inflammatory pathways activated by cytokines has been extensively studied in keratinocytes in the past [25, 26, 27], their effects on the intracellular signalling and biological functions triggered by IL-22 remain undefined. SOCS capability to regulate IL-22-induced pro-tumor responses in transformed keratinocytes is also still unknown.

In this study, we identified SOCS3 as the key SOCS family member involved in the suppression of IL-22 signaling in healthy and transformed cultured keratinocytes, and *in vivo* in a cancer mouse model of SCC. We demonstrated that SOCS3 inhibited the expression of inflammatory genes and counteracted proliferation triggered by IL-22 by down-regulating STAT3 and Erk1/2. Then, we documented that SOCS3 expression is reduced *in vivo* in BCC and SCC tumors. Interestingly, IL-22 could not induce SOCS3 expression in NMSC-derived keratinocyte lines, contrarily to that observed in healthy keratinocytes, leading to aberrant activation of STAT3 and Erk1/2, and cell proliferation and migration. Finally, the administration of a SOCS3 mimetic peptide, corresponding to KIR-ESS domain of SOCS3 opposed IL-22 effects in transformed keratinocytes *in vitro* and counteracted tumor growth in mice.

## RESULTS

### SOCS3 is strongly induced by IL-22 and inhibits IL-22-dependent STAT3 and Erk1/2 phosphorylation in human keratinocytes

**Figure 1:**
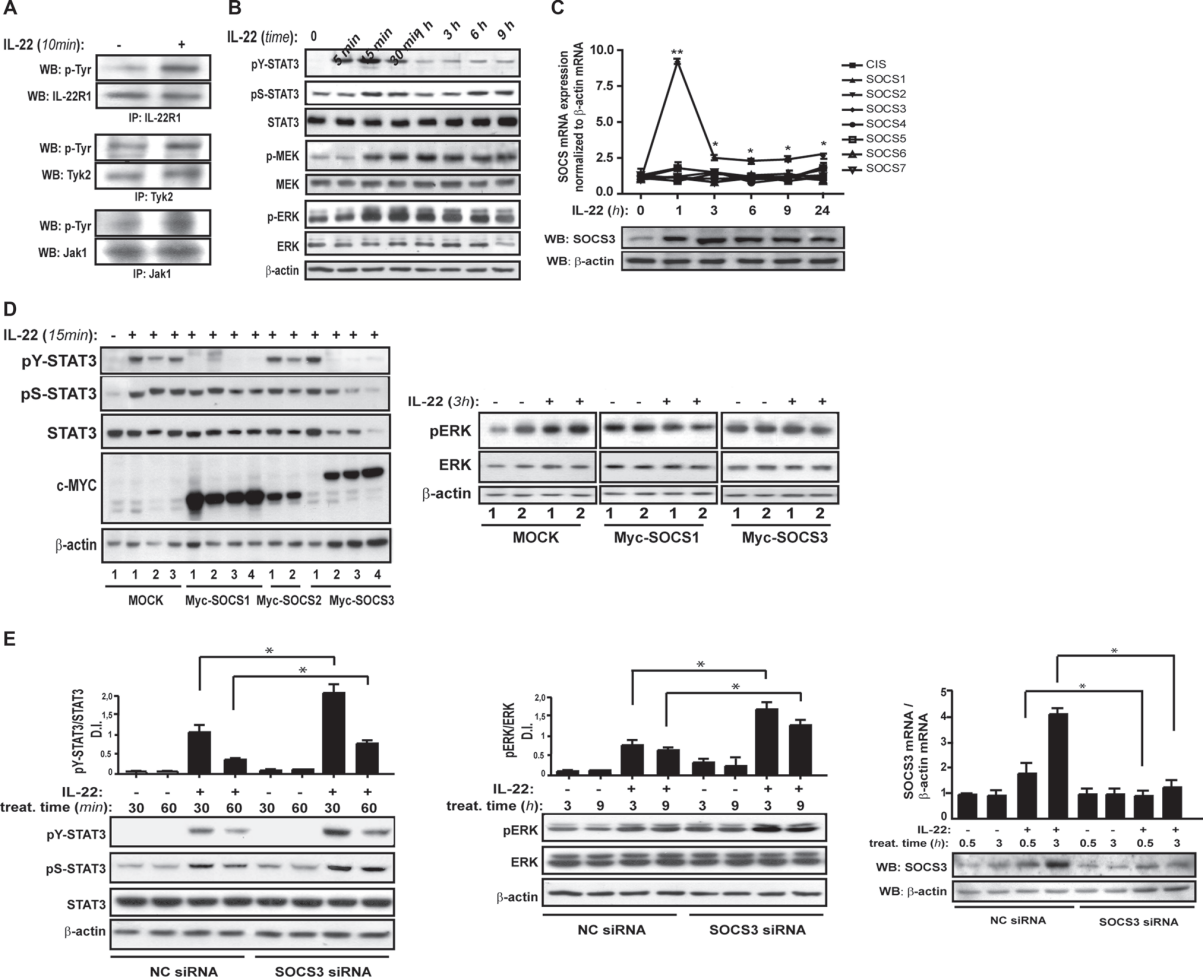
IL-22 induces SOCS3, which limits STAT3 and Erk1/2 activation (**A**) Keratinocyte cultures (*n* = 3) were stimulated with IL-22 or not for 10 min and protein lysates were subjected to immunoprecipitation and WB analyses for basal and phospho-IL-22R1, Tyk2 or Jak1. (**B**) WB of time-course experiments were performed on keratinocyte cultures (*n* = 3) to evaluate basal STAT3, Erk1/2 and MEK or phosphor-STAT3 (Tyr705 and Ser 727), phosphor-Erk1/2 and phospho-MEK. Anti-β-actin Ab was used to normalize sample loading. (**C**) SOCS mRNA and protein levels were detected by real-time PCR or WB analyses in cultured keratinocytes prepared from healthy skin (*n* = 3) left untreated or stimulated with IL-22 for the indicated time periods and normalized with β-actin values. (**D**) HaCaT cells stably transfected with myc-tagged SOCS1 (*n* = 4), SOCS2 (*n* = 2) and SOCS3 (*n* = 4) genes or control plasmids (mock, *n* = 3) were left untreated or stimulated with IL-22 for 15 min or 3 h and lysates subjected to WB with anti-phospho STAT3 (Tyr705), -phospho STAT3 (Ser727), -STAT3, -phospho Erk, -Erk, -c-myc and -β-actin Abs, as indicated. (**E**) Keratinocyte cultures were transiently transfected with siRNA specific for SOCS3 or control siRNA (NC). After 48-h transfection, cells were stimulated with IL-22 or not for the indicated time periods. In RNA interference experiments, data were obtained by three independent experiments, and SOCS3 depletion was assessed by real-time PCR and WB. Densitometric analyses were also performed on WB of STAT3 and Erk1/2 proteins. Data are expressed as ratio of densitometric intensity (D. I.) of phosphorylated and unphosphorylated STAT3 and Erk1/2 proteins. *p* < 0.05 (*) values were considered significant.

We firstly demonstrated that in human keratinocytes IL-22 induced molecular cascade implicating phosphorylation of IL-22R1, Tyk2 and Jak1 (Figure 1A), but not of Jak2 (data not shown) that culminated with STAT3, MEK1/2 and Erk1/2 activation (Figure 1B). In parallel, we observed that IL-22 also upregulated SOCS3 expression in keratinocytes, with a peak of mRNA and protein induction at 1 h and 3 h after stimulation, respectively, as demonstrated in time-course experiments (Figure 1C). SOCS3 was the only SOCS family member to be transcriptionally induced by IL-22 in keratinocytes (Figure 1C). To investigate SOCS3 effects on the IL-22-dependent signaling, keratinocyte HaCaT clones overexpressing SOCS3 were treated with IL-22 and analyzed in terms of activation of its key downstream mediators. In the same experiments, SOCS1- and SOCS2-overexpressing clones were also analyzed as keratinocytes overexpressing other SOCS family members. Contrarily to SOCS2 and mock-transfected cells, SOCS3 clones displayed total abrogation of STAT3 phosphorylation, both in Tyr705 and in Ser727 residues. Interestingly, STAT3 phosphorylation was also impaired in SOCS1-overexpressing clones (Figure 1D, left panel), even though SOCS1 was not induced by IL-22 in human keratinocytes (Figure 1C). Additionally, SOCS3 and SOCS1 clones did not show substantial upregulation of Erk1/2 phosphorylation upon IL-22 stimulation, compared to what was observed in mock-transfected clones (Figure 1D, right panel).

These data were confirmed in keratinocyte cultures transiently transfected with SOCS3 siRNA, where the effective reduction of SOCS3 levels treated or not by IL-22 was assessed both at transcriptional and protein level (Figure 1E, right panel). Consistently, SOCS3-silenced keratinocytes showed a higher STAT3 phosphorylation in Tyr705 and in Ser727 after 30–60 min of IL-22 treatment, compared to cells transfected with control siRNA (Figure 1E, left, upper panel). Also Erk1/2 was significantly increased by SOCS3 depletion (Figure 1E, left, lower panel).

As a whole, these data indicate that SOCS3 negatively regulates the intracellular pathways induced by IL-22 in human keratinocytes, by inhibiting STAT3 activation, and, in parallel, Erk1/2 phosphorylation.

### SOCS3 inhibitory action on the IL-22-dependent STAT3 activity is mediated by KIR and ESS domains

**Figure 2:**
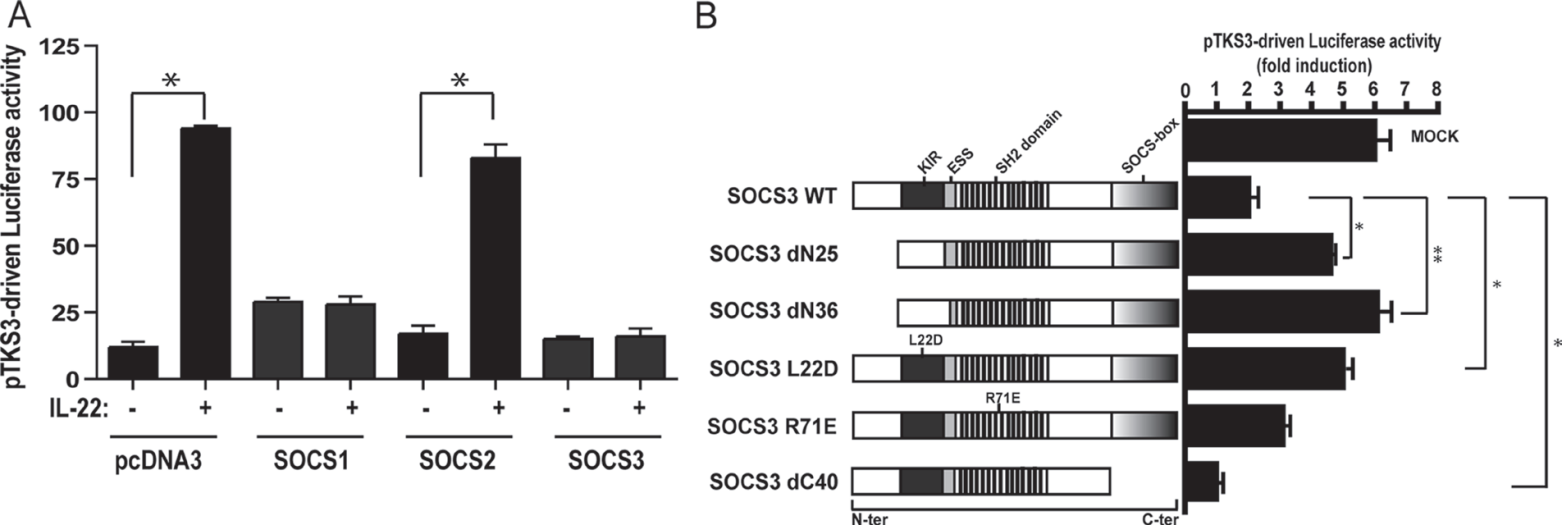
SOCS3 negatively regulates IL-22-dependent transcriptional activity of STAT3 through its KIR and ESS domains (**A**) Primary cultures of keratinocytes (*n* = 3) were transiently co-transfected with pTKS3-Luc reporter plasmid and increasing amounts pcDNA3, pcDNA3-*myc*-SOCS1, -SOCS3, or -SOCS2 cDNA containing vectors. After 18 h, transfected cells were stimulated or not with IL-22 prior to assay. B. Keratinocyte cultures (*n* = 3) were co-transfected with pTKS3 plasmid and expression plasmids carrying site-specific mutations or deleted domains of SOCS3. *Firefly* and *Renilla* luciferase activities were measured as above. All data were obtained using cellular extracts of three independent experiments, and are expressed as mean ± SD of *Firefly* luciferase values dependent on STAT3 activity normalized to *Renilla* luciferase (co-transfected control plasmid) and micrograms of proteins. In (**B**) luciferase activity of each SOCS3 plasmid was reported as fold induction between the value obtained with IL-22-treated and untreated keratinocytes. *p* < 0.05 (*) and *p* < 0.01 (**) values were significant.

To investigate the SOCS3 effects on IL-22-induced STAT3 transcriptional activity, we transiently co-transfected keratinocyte cultures with the STAT3-responsive pTKS3-Luc plasmid in presence or absence of SOCS3, SOCS1 and SOCS2 cDNA expression vectors. We found that transfection of SOCS3-, but also SOCS1-expressing plasmid, markedly reduced the transactivation of the pTKS3-Luc induced by IL-22 in treated keratinocytes, whereas SOCS2 expression or empty vectors overexpression had no effects on STAT3 activity (Figure 2A). To identify SOCS3 key domains involved in the inhibition of the IL-22-induced STAT3 activity, human keratinocyte cultures were transfected with pTKS3Luc alone or in presence of plasmids encoding different SOCS3 deletion mutants and, then, treated by IL-22. We found that the deletion of KIR region and, more markedly, of KIR added to a portion of ESS region resulted in a significant increase of the IL-22-induced pTKS3 luciferase activity, as assessed by using the SOCS3 deletion mutants dN25 and dN36, respectively (Figure 2B). Interestingly, the substitution of the leucine with aspartic acid in position 22 within KIR domain (SOCS3 L22D) strongly enhanced the pTKS3 luciferase activity compared to the wild-type SOCS3 (Figure 2B). In contrast, single R71E mutation (SOCS3 R71E) in the SH2 domain, known to abrogate the binding of SOCS3 to Jak phosphotyrosine, did not influence the inhibitory action of SOCS3 on the IL-22-induced transactivation of the STAT3-responsive plasmid. Finally, the deletion of the SOCS-box domain in SOCS3 dC40 plasmid resulted in efficient inhibition of the IL-22-mediated STAT3 activity, even at higher levels compared to the wild-type SOCS3 (Figure 2B). This latter result is likely due to SOCS3 accumulation in cells transfected with plasmid lacking SOCS box, which is known to be determinant for proteosomal-mediated degradation of SOCS molecules [17].

These data indicate that the N-terminal region overlapping KIR and ESS domains is indispensable for the inhibitory action of SOCS3 on STAT3 activity in IL-22-treated keratinocytes.

### SOCS3 counteracts STAT3-dependent gene expression and proliferation induced by IL-22 in human keratinocytes

**Figure 3:**
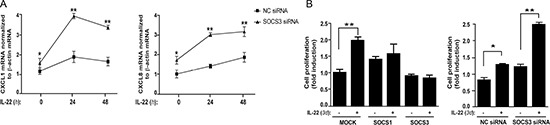
SOCS3 counteracts CXCL1 and CXCL8 expression and proliferation triggered by IL-22 in human keratinocytes (**A**) Keratinocyte cultures (*n* = 3) were transfected with SOCS3-specific or control siRNA, and, then, stimulated with IL-22 or not, for 24 h or 48 h. CXCL1 and CXCL8 mRNA levels were analyzed by real-time PCR and normalized to β-actin mRNA levels. Differences between SOCS3-silenced keratinocytes and cells transfected with control siRNA were significant. *p* < 0.05 (*) and *p* < 0.01 (**). (**B**) Proliferation of SOCS1, SOCS3 or mock clones, either in presence or absence of IL-22, was evaluated by crystal violet incorporation. This assay was also performed on keratinocytes transfected with SOCS3 or control siRNA and treated or not with IL-22. In both groups of experiments, crystal violet incorporation was measured after 3 d of culture with an ELISA reader. Values are expressed as fold induction of treated *vs* untreated samples, which were given a value of 1. *p < 0.05; **p < 0.01.

IL-22 induces effects in keratinocytes culminating with the expression of a number of molecules, such as antimicrobial peptides and proliferation-related proteins [4]. These molecules, in turn, can be inhibited by SOCS3 overexpression, as previously demonstrated in intestinal epithelial cells and in hepatocytes [28, 13]. In order to understand whether gene expression induced by IL-22 in keratinocytes could be functionally inhibited by SOCS3, we analyzed IL-22-induced genes in keratinocyte cultures silenced for SOCS3. As expected, SOCS3 depletion resulted in an higher expression of CXCL1 and CXCL8 mRNA in keratinocytes treated by IL-22, as compared to control cells (Figure 3A). On the contrary, expression of HBD-2, an antimicrobial peptide strongly induced by IL-22 in a STAT3-independent mechanism [4], was not affected by SOCS3 depletion (data not shown).

In parallel, we found that SOCS3 exerted its regulatory action also on keratinocyte proliferation triggered by IL-22. As shown in Figure 3B, the stable expression of SOCS3 and, at a lower extent, of SOCS1 significantly reduced the proliferative potential of keratinocytes under IL-22 stimulation (Figure 3B). Consistently, the SOCS3 transient depletion significantly increased their proliferative capability, as compared to control cells (Figure 3B). As a whole, these data indicate that SOCS3 negatively regulates STAT3-dependent biological effects mediated by IL-22 in human keratinocytes.

### SOCS3 is poorly expressed in non-melanoma skin tumor epidermis, and it is not induced by IL-22 in transformed keratinocytes

SOCS3 and SOCS1 expression has been widely investigated in a number of cancers, including breast cancer, multiple myeloma, hepatocarcinoma and lymphoma, where their absence suggests a tumor-suppressor function [29, 30, 31, 32]. To date, their expression in non-melanoma skin cancer remains undefined.

**Figure 4:**
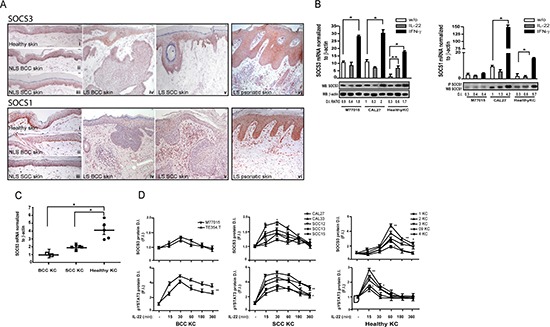
SOCS3 and SOCS1 are poorly expressed in the epidermis of NMSC lesions and are not induced in transformed keratinocytes by IL-22 (**A**) Immunohistochemistry for SOCS3 and SOCS1 (red staining) was performed respectively on frozen or paraffin-embedded sections from biopsies of healthy skin (i), NLS (ii, iii) and LS (iv, v) areas of BCC and SCC, specimens. Psoriatic skin biopsies were also analyzed (vi). Sections were counterstained with Mayer's H&E and were visually evaluated by an pathologist experienced in dermatology. One out of four representative staining is shown. Scale bars, 50 mm. (**B**) Graphs show SOCS3 and SOCS1 mRNA levels which were detected by real-time PCR analysis in cultured SCC (CAL27 and CAL33) and BCC (M77015) skin tumor lines left untreated or stimulated with IL-22 or IFN-γ for 3 h and normalized for β-actin mRNA expression levels. All data were obtained from three independent experiments. In the panel, it is also shown Western blotting (WB) or immunoprecipitation (IP) experiments performed on lysates obtained from healthy keratinocytes or M77015, CAL27 and CAL33 lines stimulated or not with IL-22 or IFN-γ for 6 h. SOCS1 was detected by IP using a specific anti-SOCS1 Ab, whereas SOCS3 by WB with an anti-SOCS3 Ab. One representative experiment of three is shown. Densitometric intensity (D.I.) of SOCS1 or SOCS3/β-actin was calculated from three independent experiments and numerically expressed as D. I. Ratio. *p < 0.01; **p < 0.05. (**C**) SOCS3 mRNA levels were detected by real-time PCR analysis in BCC (*n* = 2), SCC lines (*n* = 5) and healthy keratinocyte strains (*n* = 5), left untreated or stimulated with IL-22 for the optimal treatment period to induce SOCS3 (i. e. 1 h for healthy cells and 15 min for transformed cell lines) and normalized for β-actin mRNA expression levels. Data are reported as fold induction (F. I.) between values obtained from IL-22-treated and untreated cells. * *p <* 0.01. (**D**) BCC (*n* = 2), SCC lines (*n* = 5) and healthy keratinocyte strains (*n* = 5) were left untreated or stimulated with IL-22 for the indicated time periods. SOCS3 and phospho-Tyr STAT3 levels were detected by WB and normalized to β-actin levels. Data represent the F. I. of the D. I. values between IL-22-treated and untreated samples, to which a value of 1 was given. *p < 0.05; **p < 0.01.

To this end, we analyzed the *in vivo* expression of SOCS3 and SOCS1 in BCC or SCC skin tumor specimens, including lesional (LS) and non-lesional (NLS) zones. The quantifications of SOCS3 and SOCS1 immunoreactivities in the epidermis of SCC and BCC skin tumors were evaluated by using a four-stage scoring system, and as shown in Figure 4A, a weak cytoplasmic expression of SOCS3 was observed in the epidermis of both NLS BCC and SCC skin, as well as of healthy skin (all specimens were scored 1+). Interestingly, SOCS3 staining remained quite absent in the epidermal layers of LS BCC specimens (score 1+), whereas it was moderate in the epidermal compartment of LS SCC skin (score 2+). In both skin tumors, SOCS3 was not detectable in immune cells infiltrating the dermis. SOCS1 immunoreactivity was present in the epidermal basal layers of NLS zones of BCC and SCC tumors or in healthy skin, even though it was detected in the nuclear compartment (Figure 4A, all specimens were scored 1+). SOCS1 expression completely disappeared in epidermal layers of LS BCC specimens (score 0), and it was only weakly expressed in the epidermal keratinocytes of LS SCC skin (score 1+). In both skin tumors, a high number of infiltrating immune cells expressing SOCS1 was detectable. Of note, SOCS3 and SOCS1 expression observed in LS BCC and LS SCC was lower than that present in LS psoriatic skin, where it abundantly accumulates, in particular in the cytoplasmic compartment, as a consequence of the establishment of an inflammatory cytokine milieu by infiltrating immune cells (Figure 4A, score 3+).

Next, we analyzed SOCS3 and SOCS1 levels in SCC and BCC skin tumor lines left untreated or stimulated with IL-22. In parallel, these cell lines were treated with IFN-γ, the most potent cytokine able to induce SOCS1 and SOCS3 in healthy keratinocytes. We found that IL-22 significantly induced mRNA and protein expression of SOCS3 in healthy keratinocytes, but not in SCC or BCC tumor cells (Figure 4B). However, SOCS3 and SOCS1 were significantly up-regulated by IFN-γ in all cell lines both at transcriptional and protein level, with the exception of SOCS1 protein in the BCC lines (Figure 4B). The weak SOCS3 mRNA induction by IL-22 was confirmed in all the cancer cell lines examined (*n* = 2 for BCC and *n* = 5 for SCC), as compared to healthy keratinocyte strains (*n* = 5) (Figure 4C). As possible consequence of this weak SOCS3 induction observed in transformed cells in all time-points of IL-22 stimulation (Figure 4D), phospho-STAT3 levels were higher compared to healthy cells, and showed a long-lasting activation (Figure 4E).

Taken together, these data indicate a dysregulated expression of SOCS3 in transformed keratinocytes upon IL-22 exposure.

### A SOCS3-derived peptide prevents IL-22-induced STAT3 and Erk activation in transformed keratinocytes

Starting from structural and biochemical studies of SOCS3/Jak2 complex [18], we developed two SOCS3-derived peptides, named KIR and KIR-ESS, spanning the aminoacidic region from 22 to 33 and from 22 to 45, respectively (Table 1). These peptides were able to bind *in vitro* Jak2 and Tyk2, provinding, for KIR-ESS, K_D_ values of 33 and 40 μM, respectively (unpublished data). As controls, two peptides overlapping KIR-ESS sequence, but modified in residues crucial for binding to Jaks, were designed (Table 1).

**Table 1:** List of peptides used in the study^a^

Peptides	^N-ter^Sequences ^C-ter^
**Ctrl1**	^22^LKTFSSKSAYQAAANAARAAQEAG^45^
**Ctrl2**	^22^LKTFSSKSEYQLAANAARAAQEAG^45^
**KIR**	^22^LKTFSSKSEYQL^33^
**KIR-ESS**	^22^LKTFSSKSEYQLVVNAVRKLQESG^45^

*^a^*SOCS3-derived sequences were employed conjugated to a cell penetrating peptide from TAT-sequence to their N-terminus for delivery in the cells. Ctrl1 and Ctrl2, control peptides, are identical in aminoacid sequence to KIR-ESS peptide with the exception of the underlined residues (Ala substitution).

**Figure 5:**
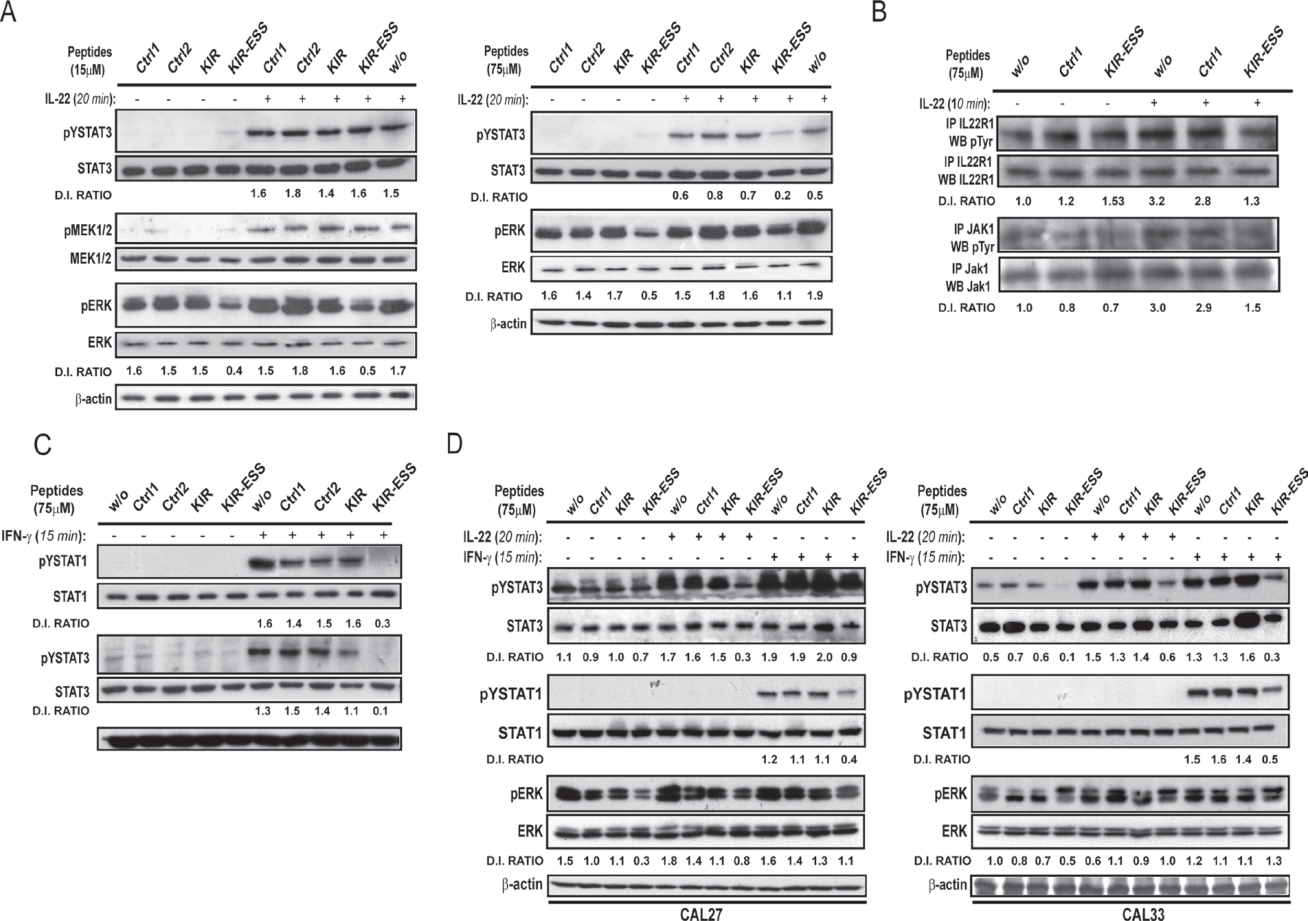
KIR-ESS peptide inhibits IL-22-induced phosphorylation of STAT3 and Erk in healthy and SCC-derived keratinocytes (**A**) Protein extracts obtained from healthy keratinocytes (*n* = 3) pre-treated or not with 15 μM (left panel) or 75 μM (right panel), KIR, KIR-ESS and control (Ctrl1 or Ctrl2) peptides, and, then, stimulated or not with IL-22 for 20 min, were subjected to WB analysis to detect STAT3 (Tyr705), Erk and MEK phosphorylation. Filters were probed with anti-STAT3, -Erk and –MEK Abs (top). (**B**) Protein extracts obtained from healthy keratinocytes (*n* = 3) pre-treated or not with 75 μM KIR-ESS and control (Ctrl1) peptides, and, then, stimulated or not with IL-22 for 10 min, were subjected to immunoprecipitation for IL-22R1 and Jak1, and then analysed by WB to detect phosphorylated and unphosphorylated forms of the proteins. (**C**) Unphosphorylated and phosphorylated forms of STAT1 (Tyr701) and STAT3 (Tyr705) were monitored in keratinocytes treated with IFN-γ for 15 min by WB analysis (bottom). In all experimental groups, filters were stripped and re-probed with β-actin Ab. (**D**) Protein extracts were obtained from CAL27 (left panel) or CAL33 (right panel) lines treated or not with 75 μM peptides in presence of IL-22 or IFN-γ. WB was performed to detect basal and phosphorylated STAT1, STAT3 and Erk. D.I. ratio indicates the densitometric intensity of phosphorylated/unphosphorylated target protein.

Thus, we investigated the KIR and KIR-ESS peptide capability of abrogating IL-22-mediated intracellular signaling in human keratinocytes. To this end, keratinocyte cultures were pre-treated with SOCS3 or control (Ctrl1 or Ctrl2) peptides, and then stimulated with IL-22. We found that a low dose of KIR-ESS, but not of KIR, significantly reduced Erk1/2 phosphorylation in both untreated and IL-22-treated cultures, whereas they did not affect STAT3 phosphorylation (Figure 5A, left panel). However, a higher dose of KIR-ESS reduced not only the phosphorylation of Erk, but also of STAT3 (Figure 5A, right panel). KIR-ESS high-dose treatment also impaired the phosphorylation of IL-22R1 subunit and Jak1 (Figure 5B), whereas it seemed to not affect Tyk2 phosphorylation (data not shown). Interestingly, MEK phosphorylation was not affected by KIR-ESS treatment, indicating that the peptide directly inhibits Erk1/2 phosphorylation (Figure 5A).

Due to its KIR domain, SOCS3 is known to suppress the activation not only of Jak1 and Tyk2, but also of Jak2, a pivotal kinase of the IFN-γ-dependent signaling [16]. Thus, we investigated whether KIR-ESS and KIR were able to inhibit IFN-γ-induced pathway, which activates the transcriptional factors STAT1 and STAT3 following to Jak1 and Jak2 phosphorylation. To this end, keratinocyte cultures were pre-treated with KIR-ESS, KIR or control peptides, and, then, stimulated with IFN-γ for 15 min. We found that KIR-ESS totally abrogated the IFN-γ-dependent phosphorylation of STAT1 in Tyr701 and STAT3 in Tyr705. KIR peptide also reduced STAT1 and STAT3 activation even though less efficiently that KIR-ESS (Figure 5C).

It has been recently demonstrated that, STAT3 is constitutively activated in BCC or SCC-cell lines and it is strongly upregulated by IL-22 [10]. Erk1/2 are also constitutively phosphorylated in SCC-cell line, even though cannot be activated by IL-22 in CAL33 and only slightly upregulated in CAL27 [10]. Therefore, we investigated the effects of KIR-ESS and KIR peptides on the activation of STAT3 and Erk1/2 in SCC and BCC lines.

As shown in Figure 5D, we found that KIR-ESS, but not KIR, strongly reduced STAT3 phosphorylation in both untreated and IL-22-treated CAL27 (left panel) or CAL33 cell lines (right panel). STAT1 and STAT3 phosphorylation induced by IFN-γ was also impaired by KIR-ESS in both cell lines. In addition, KIR-ESS peptide significantly reduced Erk1/2 phosphorylation in both untreated and IL-22-treated CAL27, whereas its effect was less evident upon IFN-γ exposure (Figure 5D, left panel). In CAL33, KIR-ESS reduced only moderately phosphorylated Erk1/2 total levels, with a predominant inhibitory effect on Erk2 (Figure 5D, right panel). Finally, KIR-ESS peptide did not influence STAT3 and Erk1/2 phosphorylation in BCC line treated with IL-22 or IFN-γ (data not shown). As a whole, these data indicate that KIR-ESS peptide prominently reduces the intracellular signaling induced not only by IL-22 but also by IFN-γ in both healthy and transformed keratinocytes.

### SOCS3-derived KIR-ESS peptide dampens the proliferative and migratory potential of transformed keratinocytes

**Figure 6:**
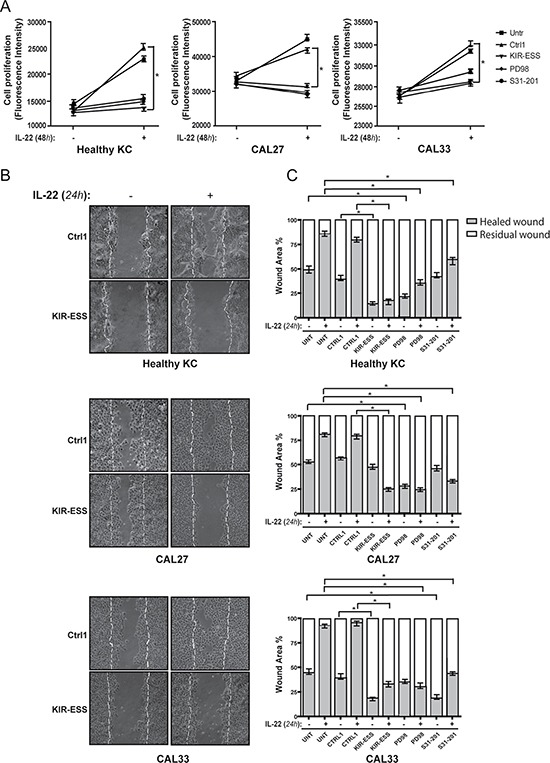
IL-22-induced proliferation and migration of healthy and transformed keratinocytes is counteracted by KIR-ESS peptide (**A**) Healthy KC, CAL27 and CAL33 cultures were plated in 96-well plates in quadruplicate for each condition. After 1 day, cells were pre-treated or not with Ctrl1 or KIR-ESS peptides (40 μM), or S31-201 (10 mm) and PD98059 (20 mm) chemical inhibitors, and, then, stimulated or not with 50 ng/ml of IL-22. Data relative to 48-h treatment are shown and expressed as the values of fluorescence intensity obtained from untreated and IL-22-treated ± SD. Three independent experiments were performed. *p < 0.01. (**B**) Scratch assays were carried out on healthy or SCC-lines, pre-incubated with mitomycin C (5 mg/ml), pre-treated with Ctrl1 or KIR-ESS peptides (40 μM), or S31-201 (10 mm) or PD98059 (20 mm) chemical inhibitors, and, then, stimulated or not with IL-22 for 24 h. Microscopic images were taken immediately after and 24 h after wound induction on confluent cell layers. Initial scratches (0 h) are dashed blank. (**C**) Cell-free area was measured and indicated as residual wound. Data are reported as healed wound (gray area of bars) *vs* residual wound (white area of bars) which a value of 100% was given. *p < 0.01.

IL-22 promotes tumorigenic functions, by activating STAT3 phosphorylation and inducing mitogenic and other pro-tumor genes in several tumor lines, including BCC and SCC lines [33, 11]. Therefore, we investigated whether KIR-ESS peptide could affect the biological functions triggered by IL-22 in keratinocytes. To this end, healthy keratinocytes and SCC-cell lines treated or not with KIR-ESS or Ctrl1 peptides were left untreated or stimulated with IL-22, and, then, analyzed in terms of proliferation and migration. The optimal concentration of KIR-ESS or Ctrl1 peptides used in these experiments was determined in healthy and transformed keratinocyte cultures by evaluating the best ratio between null cellular toxicity and efficacy at long-term peptide treatment (data not shown). As shown in Figure 6A, similarly to Ctrl1, KIR-ESS peptide did not significantly influence the proliferation of healthy and transformed keratinocytes in absence of IL-22. However, KIR-ESS significantly impaired the proliferative response to IL-22 not only of SCC-cell lines but also of healthy keratinocytes. KIR-ESS effect was similar to that observed in all healthy and transformed keratinocyte cultures treated by the chemical Erk1/2 and STAT3 inhibitors, PD98059 and S3I-201, respectively. Furthermore, we analyzed the effects of KIR-ESS peptide on the migration of transformed or healthy keratinocytes in an *in vitro* skin injury model. KIR-ESS hindered the closure of wounds in IL-22-treated SCC-scratched cultures, with an effect similar to those obtained with the chemical Erk1/2 and STAT3 inhibitors, PD98059 and S3I-201, respectively (Figure 6, panels B and C). KIR-ESS inhibitory effect on wound closure was also observed in untreated cells, with the exception of CAL27 cell line. In parallel, the employment of S31-201 or PD98059 inhibitors on resting cells was efficacious in inhibiting migration of CAL27 cells and healthy or CAL33 keratinocytes, respectively (Figure 6C). These data indicate that KIR-ESS peptide likely inhibits STAT3-dependent pro-migratory function in CAL33 cells and Erk1/2-dependent migration of healthy keratinocytes or CAL27 transformed cells.

These data demonstrate the efficacy of KIR-ESS in inhibiting IL-22 tumorigenic functions (proliferation and migration), and confirm the key role of both STAT3 and Erk1/2 in such processes.

### KIR-ESS peptide inhibits tumor growth *in vivo*

**Figure 7:**
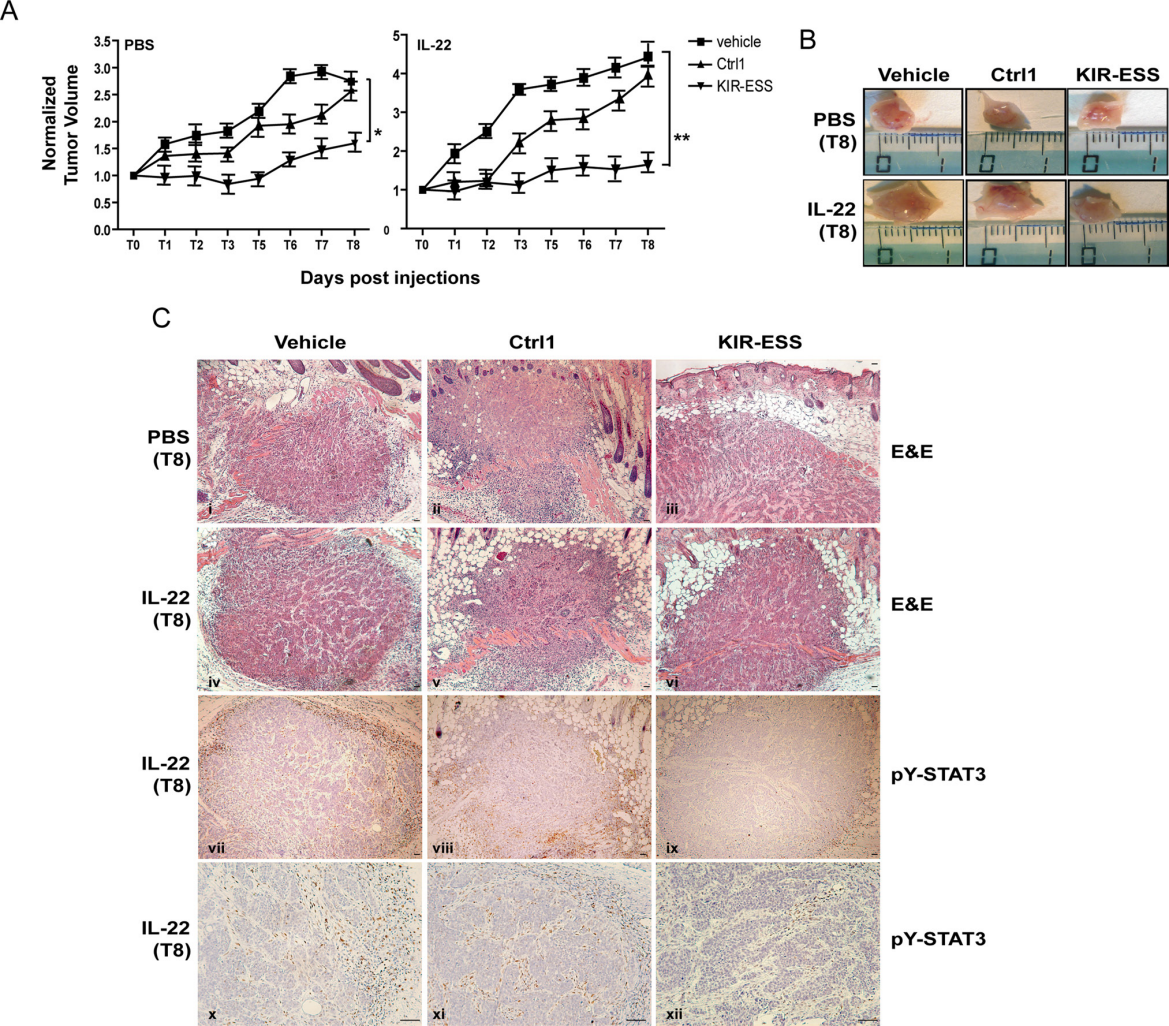
KIR-ESS efficiently targets STAT3 phosphorylation in SCC xenografts and inhibits tumor growth in nude mice (**A**) CAL27 cells (5 × 10^6^) were subcutaneously injected in athymic 8 week old, male, nude mice. Once tumors were established, mice (5/group) were randomized to receive injections of 330 ng of IL-22 or 1× PBS once a day for 7 days on the tumor boundaries, in presence of peptide vehicle (1 × PBS), Ctrl1 or KIRESS (100 μg). Tumor volumes were calculated daily as indicated in Material and Method, normalized to the volume at first day of treatment and plotted along the Y axis for PBS- (left panel) or IL-22- (right panel) treatment. Comparison was done by *t* test (*p < 0.05; **p < 0.01). (**B**) Representative images of xenografts at T8 of each PBS- or IL-22-treated group (vehicle, Ctrl1 and KIR-ESS) are shown. (**C**) Representative images of paraffin-embedded sections obtained from tumor biopsies were counterstained with Mayer's H&E (i-vi) or stained for pY-STAT3 (vii–xii) with specific anti-pY-STAT3 Ab (brown staining). Scale bars: 100 μm.

To evaluate whether KIR-ESS peptide could prevent tumor growth *in vivo*, we generated tumor xenografts in athymic nude mice by injecting CAL27 cell line subcutaneously in the animal back. Once tumor were established, mice were subjected or not to subcutaneous injections of IL-22 once a day for 7 days, in presence of peptide vehicle, Ctrl1 or KIRESS peptides. We observed that in absence of IL-22, mice treated with KIR-ESS peptide displayed reduced tumor volumes compared with those treated with Ctrl1 peptide or vehicle alone, reaching a final volume of 2.7 ± 0.15 cm^3^ for vehicle or 2.4 ± 0.18 cm^3^ for Ctrl1 groups and 1.5 ± 0.14 cm^3^ for KIR-ESS group (*p* < 0.05; Figure 7A). The reduction of tumor growth due to KIR-ESS treatment was even more evident when IL-22 was injected in tumor boundaries, with a final volume of 1.5 ± 0.16 cm^3^ for KIR-ESS group, and 4.3 ± 0.16 cm^3^ for vehicle or 3.9 ± 0.16 cm^3^for Ctrl1 group (*p* < 0.01; Figure 7A). At day 8, mice were sacrificed, and tumors measured and processed for histological and IHC analyses (Figure 7B and 7C). As shown in Figure 7C, we could not observe significant structural differences in tumor histological organization, with untreated or IL-22-treated tumors showing the presence of proliferating (Ki-67^+^, Keratin 14^+^ and Keratin 5^+^) and differentiating (Keratin 1^+^) keratinocytes in tumor outer or inner cell masses, respectively (data not shown), as previously reported [34]. Interestingly, treatment with KIR-ESS peptide significantly reduced pY-STAT3 expression levels in both untreated and IL-22-treated SCC xenografts, in particular in those areas surrounding tumor mass (Figure 7C and data not shown) likely enriched of keratinocytes responsible for maintaining tumor growth [34]. In addition, pY-STAT3 positivity was also reduced in areas within tumors where fibrotic tissue accumulates between SCC keratinocyte islets (Figure 7C). Total pY-STAT3^+^ cells were reduced by 75 % or 65% in tumors co-treated with IL-22 and KIR-ESS peptide, as compared to treatments with vehicle or Ctrl1 peptide, respectively.

## DISCUSSION

NMSC is the most prevalent cancer in light-skinned populations, and mainly includes BCC, representing around 75% of NMSC, and SCC [35]. In the past, molecular pathways critically involved in the development of BCC and SCC, including EGFR/ERK and IGFR signalings, have been identified, leading to new pharmacological approaches for the treatment of these skin tumors [36, 37].

In this study, we focused on IL-22 signaling and identified SOCS3 protein, an intracellular inhibitor of cytokine-associated signaling, as a valid candidate to suppress tumor responses of BCC and SCC. The attempt of counteracting IL-22 effects in transformed keratinocytes arises from recent studies from our laboratory showing that IL-22 is abundantly secreted by TILs infiltrating BCC and SCC lesions, and, together with IL-17, it displays has tumorigenic functions in these pathological contexts [10]. In particular, IL-22 promotes proliferation and migration of BCC and SCC cell lines, as well as tumor growth in an *in vivo* xenograft model [10]. These effects are associated with the induction of IL-6 and CXCL8, two cytokines known to enhance tumor progression and to be also produced in other tumor contexts, including such as ovarian, prostate and breast cancers, as well as hepatocellular, esophageal and gastric carcinoma [38, 39, 40]. Interestingly, IL-22 stimulation of BCC and SCC lines resulted in the activation of Erk1/2, STAT3 and Akt molecules, suggesting that these pathways may be responsible for the proliferative effects mediated by IL-22 observed both *in vivo* and *in vitro* [10].

In this study, we tested the effects of a newly-developed peptide, mimicking KIR and ESS domains of SOCS3 protein, in cultured transformed and healthy keratinocytes, following IL-22 stimulation, and in a cancer mouse model of SCC. We found that *in vivo* tumor growth was potently reduced by KIR-ESS administration, especially when xenografts were subjected to IL-22 treatment. The capability of KIR-ESS to inhibit tumor growth correlated with its efficacy in reducing levels of pY-STAT3 within the tumors, particularly in those areas surrounding tumor mass likely enriched of keratinocytes responsible for maintaining tumor growth [34]. At molecular level, we identified STAT3 and Erk1/2 as key intracellular targets of KIR-ESS peptide, since their phosphorylation was strongly reduced by its administration. We suppose that in both healthy and tumor contexts, this effect depends on the impaired phosphorylation of molecules involved in the IL-22 proximal signaling, such as IL-22R1 and Tyk2. As a direct consequence of the impaired activation of key IL-22 signaling mediators, KIR-ESS peptide strongly counteracted the proliferative and migratory potential NMSC-derived keratinocytes promoted by IL-22. KIR-ESS inhibitory effect on migration was also observed in absence of IL-22, although not in all keratinocyte cell lines examined. Similarly, the employment of S31-201 (STAT3 inhibitor) or PD98059 (Erk1/2 inhibitor) could have or not inhibitory effects depending on keratinocyte cell strains examined. These data indicate that KIR-ESS peptide can inhibit both STAT3-dependent and Erk1/2-dependent pro-migratory mechanisms in untransformed and transformed keratinocytes, depending on STAT3 and/or Erk1/2 expression levels.

Our attempt to inhibit the IL-22 signaling by reinforcing the inhibitory SOCS3 action was supported by our observation that SOCS3 is poorly expressed in the BCC and SCC tumor lesions. Interestingly, we found that, in contrast to what we observed in healthy keratinocytes, IL-22 could only weakly induce SOCS3 expression in transformed BCC and SCC keratinocytes. In parallel, pY-STAT3 levels were higher compared to healthy cells, both at basal level and after IL-22 treatment, and showed a long-lasting activation. Therefore, it is likely that STAT3 hyperactivation in BCC or SCC-cell lines is a consequence of their reduced capability to express SOCS3 in response to IL-22.

SOCS3 expression moderately detected *in vivo* in BCC and SCC lesions can be influenced by the complex tumor microenvironment, where other cytokines, such as IL-6, could induce SOCS3 *via* STAT3. In addition, we found that IFN-γ induced SOCS3 at high levels in transformed keratinocytes *in vitro*, indicating that the impaired expression of SOCS3 in these cells depends on the specific stimulus. Indeed, tumor cells can develop resistance mechanisms that favour cancer growth, and the compromised expression of SOCS3 in response to IL-22 might be representative of this situation. Of note, TILs infiltrating NMSC lesions produce quite very low levels of IFN-γ [10]. In addition, we demonstrated that also SOCS1 expression is significantly reduced in NMSC lesions. These results fit with previous observations showing a deficit of SOCS1 and SOCS3 in various tumor contexts, including prostate cancer [41], multiple myeloma [30], hepatocellular carcinoma [31] and lymphoma [32]. Such a decrease, commonly associated with a transcriptional silencing or a selective unresponsiveness to a stimulus, is reasonable determined mostly by an aberrant promoter methylation of SOCS genes [19]. In some cases, the lack of SOCS1 and SOCS3 contributes to the growth of tumor mass induced by cytokines or growth factors, thus confirming the tumor suppressor function of these two molecules. Consistently, hyper-methylation of the SOCS3 promoter is found in 90% of head and neck cancer [42], followed by lung cancer, prostate cancer, Barrett esophagus carcinoma and ulcerative colitis-related colorectal cancer [19], suggesting that methylation-induced inactivation of the SOCS3 gene may be an early event in these cancers. DNA hypermethylation of SOCS1 is also frequently found in certain types of lymphomas and in myelodysplastic syndrome, which may result in enhanced STAT1 and Jak2 activity and hence cell proliferation [43, 44]. SOCS3 deficiency indirectly sustains tumorigenesis by aberrantly activating STAT3, which promotes cell cycle and suppresses anti-apoptotic events. Potential target genes of STAT3 are cell survival genes, including Bcl-2 and Bcl-xL, and cell cycle regulators, such as cyclin D1 and cyclin E1, and p21 [45]. VEGF, which is among the target of STAT3, also contributes to tumor angiogenesis [46]. Moreover, STAT3 is involved in suppression of p53 expression and p21, a p53 target gene [47, 48]. However, recent studies have demonstrated that SOCS1 could be directly implicated in tumor suppression by blocking mitosis in melanoma cell lines. In particular, SOCS1 was shown to reduce protein levels of G1 phase regulators of cell-cycle, such as cyclin D and cyclin E levels, and alters M phase protein levels through Cdh1 degradation [49]. Evidence of a direct role of SOCS3 in tumor progression in terms of disruption of cell cycle does not exist, likely due to the fact that SOCS3, differently from SOCS1, cannot localize in the cell nucleus, since it lacks of a nuclear translocation domain [50]. Finally, in this study we showed that other than SOCS3, SOCS1 forced expression was also effective in countering IL-22-mediated responses in human keratinocytes, even though it could not be directly induced by IL-22. This inhibition could be functionally explicated by the KIR region of SOCS1 molecule [17]. Since SOCS1 is also not adequately expressed in BCC and SCC lesions, it would be interesting to evaluate the potential anti-tumor effects of KIR-SOCS1 mimetic peptides, such as PS5, in BCC and SCC lines.

As a whole, these data provide a rationale for the use of peptides mimicking the action of SOCS3 in the therapy of skin BCC and SCC tumors, since they are able to inhibit the deleterious effects of IL-22 in these diseased contexts.

## MATERIALS AND METHODS

### Patients and ethics statement

Skin tumor biopsies were obtained from patients affected by BCC (*n* = 8) or SCC (*n* = 10) undergoing surgical intervention to remove skin cancer lesions at IDI-FLMM. *n* = 5 patients affected by plaque-type psoriasis were also included in the study. Human keratinocyte cultures were established from the skin of healthy subjects undergoing plastic surgery. Primary cultures of human keratinocytes were prepared as previously described [27], and harvested in aliquots in nitrogen liquid at −180°C, in the cell bank of the Laboratory of Experimental Immunology of IDI-FLMM. Patients were enrolled in the study after written informed consent. This study was approved by the institutional review boards of the IDI-FLMM and were conducted according to the Declaration of Helsinki Guidelines.

### SOCS3 mimetic peptides

The SOCS3 mimetic peptides, named KIR or KIR-ESS, were designed starting from structural and biochemical studies of SOCS3/Jak2 complex [18]. Control peptides (Ctrl1 and Ctrl2) were identical to SOCS3 KIR-ESS region with aminoacid substitutions in positions critical for SOCS3 and Jak2 interactions (Table 1). Peptides were synthesized through solid phase peptide synthesis, performed on a fully automated multichannel peptide synthesizer Syro I (Multisynthech, Witten, Germany). Preparative RP-HPLC was carried out on a Shimadzu LC-8A, equipped with a SPD-M10 AV detector and a Phenomenex C18 Jupiter column (50 × 22 mm ID; 10 μm) (Shimadzu, Japan). Peptides were then, analyzed by mass spectrometry, carried out on an LCQ DECA XP Ion Trap mass spectrometer equipped with an OPTON ESI source, operating at 4.2 kV and 320°C, and with a complete Surveyor HPLC system (ThermoFisher Scientific, Waltham, MA USA). To facilitate the peptide delivery into cell cytoplasm, the fragment 48–60 of the HIV Tat protein was conjugated to SOCS3 or to control peptides in a stepwise manner. Purified peptides were lyophilized and stored at −20°C until use.

### Immunohistochemistry

Tumor or psoriatic skin tissues were harvested and divided in two parts, which were either fixed with 4% paraformaldehyde or frozen in OCT compound. 5-μm paraffin-embedded sections were dewaxed and rehydrated whereas cryostatic sections were fixed with cold acetone. Paraffin-embedded or cryostatic sections were incubated with primary mAbs, anti-SOCS1 or anti-SOCS3, respectively (both from MBL International Corporation, Nakaku Nagoya, Japan), for 1 h at room temperature. Secondary biotinylated mAbs and staining kits (Vector Laboratories, Burlingame, CA, USA) were used to develop immunoreactivities. Sections were counterstained with Mayer's H&E and were visually analyzed by two pathologist experienced in dermatology and positivity was evaluated in 5 adjacent fields at a magnification of 200×. A semiquantitative, four-stage scoring system was applied, ranging from negative immunoreactivity (0) to strong immunoreactivity (3+) for SOCS1 or SOCS3 in the epidermis.

### Keratinocyte cultures and treatments

2nd- or 3rd-passage cultures of human keratinocyte strains (*n* = 5), obtained as previously described [4, 14], were grown in serum-free Keratinocyte Growth Medium (KGM; Clonetics, Walkersville, MD, USA), for 3–5 days (at 60–80% confluence) before performing experiments. The HaCaT human keratinocyte cell line was a gift from N.E. Fusenig (Deutsches Krebsforschungszentrum, Heidelberg, Germany), and was grown in Dulbecco modified Eagle medium (DMEM; Biochrom, Cambridge, UK) supplemented with 10% Fetalclone II serum (HyClone Laboratories, South Logan, UT, USA). SCC-derived cell lines, CAL27-and CAL33 (American Tissue Culture Collection, ATCC, Milan, Italy), SCC12, SCC13 and SCC-15 (kindly provided by Dr. A. Marconi, University of Modena e Reggio Emilia, Italy), as well as M77015-08 and TE354.T (ATCC) were grown in DMEM (Biocrhom AG, Cambridge, UK) with 10% fetal bovine serum (HyClone Laboratories). Primary cultures of healthy keratinocytes and skin tumor cell lines were treated with peptides (KIR, KIR-ESS, Ctrl1 and Ctrl2) resuspended in 1X PBS for 1 h and, then, stimulated with 50 ng/ml IL-22 or 200 U/ml IFN-γ (R&D Systems, Minneapolis, MN, USA) in keratinocyte basal medium (KBM, Clonetics) or DMEM, respectively. Peptide concentrations used for these studies were accurately selected for their low cytotoxic effects on keratinocyte cultures (data not shown).

### Western blotting and immunoprecipitation

Total protein extracts were prepared as previously reported [27]. Immunoprecipitation and immunoblotting were performed accordingly to standard procedures [27]. The Abs employed for the study were as follows: anti-IL-22R1, anti-phospho-STAT3 (Ser727), anti-STAT3 (C-20), anti-phospho-STAT1 (Tyr701), anti-STAT1 (E-23), anti-phospho-Erk1/2 (E-4), anti-Erk1/2 (C16), anti-β-actin (C-11), and HRP-conjugated anti-c-myc (9E10), all provided by Santa Cruz Biotechnology (Santa Cruz, CA). Anti-phospho-STAT3 (Tyr705) anti-MEK (mitogen-activated protein kinase) 1/2 and anti-phospho MEK1/2 (Ser218/222) were from Cell Signaling Technology (Denvers, MA); anti-SOCS1 and anti-SOCS3 were from MBL (Sigma-Aldrich, Milan, Italy), whereas anti-JAK1 and anti-phospho Tyr were from Upstate (Millipore, Milan, Italy). Filters were properly developed with anti-mouse, anti-goat, or anti-rabbit Ig Abs conjugated to HRP using the ECL-plus detection system (Amersham, Dubendorf, Switzerland), or, otherwise, the SuperSignal West Femto kit (Pierce, Rockford, IL, USA).

### RNA isolation, polymerase chain reaction (PCR) and real-time PCR

Total RNA isolation and real-time RT-PCR analyses for SOCS mRNA expression were performed as previously described [27]. The forward and reverse primers employed were as follows: for SOCS1, 5′-TTT TTCGCCCTTAGCGTGA-3′ and 5′-AGCAGCTCGAAG AGGCAGTC-3′; for SOCS3, 5′-AAGGACGGAGACTTC GATTCG-3′ and 5′-AAACTTGCTGTGGGTGACCAT-3′; for CXCL8, 5′-GCTGGCTTATCTTCACCATCATG-3′ and 5′-TTATTTTTTTTCAGTTAATTAACAGATGCT ATCAT-3′; and for β-actin, 5′-CATCGAGCACGGCATC GTCA-3′ and 5′-TAGCACAGCCTGGATAGCAAC-3′. The sequences of the primers and internal probe for HBD-2 mRNA have been previously described [4]. Primers for CIS (Hs003203371), SOCS2 (Hs00919620), SOCS4 (Hs00328404), SOCS5 (Hs00367107), SOCS6 (Hs00377781), SOCS7 (Hs00389987), CXCL1 (Hs00236937), and HPRT1 (Hs01003267) were purchased by Applied Biosystems (Branchburg, NJ, USA). Fluorescence intensity was analyzed by the ABI PRISM SDS 7000 PCR Instrument (Applied Biosystems). The fold induction value for triplicate wells was presented as the mean ± S.D.

### Transient RNA interference

SOCS3 was knocked-down by using a pool of 4 small short interfering (si)RNA ON-TARGET *plus* SMARTpool (L-004299-00-0005, Dharmacon RNA Technology, Lafayette, CO, USA). In parallel, a pool of 4 non-targeting siRNA was used as negative control (L-011511-00-0005). Primary cultures of keratinocytes were transfected with SOCS3 or control siRNA at 50 nM final concentration, using Interferin reagent (Polyplus Transfection, New York, NY, USA). After 2 days of transfection, keratinocytes were treated with IL-22 for the indicated time periods.

### Transient transfections of keratinocytes and luciferase assay

Cultured keratinocytes grown in 12-well plates were transiently transfected with the STAT3-responsive pLucTKS3 plasmid (a generous gift of Prof. J. Turkson, University of Central Florida, Orlando, FL, USA), by using Fugene reagent (Promega, Madison, WI, USA), as previously reported [26]. After transfection, cells were stimulated with IL-22 for 8 hours, and *Firefly* luciferase activity was measured using the Dual-Glo Luciferase Assay System (Promega). pRL-null plasmid encoding the *Renilla* luciferase was included in each transfection. Alternatively, cultured keratinocytes were transiently transfected with pcDNA-myc/SOCS1-2-3 (a generous gift of Prof A. Yoshimura, Dept. of Microbiology and Immunology, Keio University, Japan) and pLucTKS3 plasmid or pcDNA3.1 plasmid, with the latter being used as negative control. Additionally, a panel of plasmids expressing wild-type or mutated SOCS3 was transiently co-transfected with the pTKS3Luc plasmid in cultured keratinocytes, and luciferase activity was evaluated after IL-22 treatment. SOCS3 mutated plasmids, such as dN25, dN36, L22D, R71E and dC40 were a generous gift of Prof. A. Yoshimura.

### Stable transfection

HaCaT cells were permanently transfected with pcDNA-myc/SOCS1–3 or empty pcDNA3 plasmids, as previously described [25]. HaCaT clones expressing SOCS molecules were selected by Western blot analysis using anti-c-*myc* 9E10 mAb (Santa Cruz, CA).

### Cell proliferation analysis

SOCS1- and SOCS3 stably transfected keratinocytes or cultured keratinocytes previously transfected with SOCS3 or control siRNA were seeded in 96-well plates (1.5–2.0 × 10^4^ cells/well), and, the day after, starved in medium without serum. At 60% confluence, cells were treated with 75 ng/ml of IL-22 for 3 days, stained with 0.5% crystal violet (Bio-Rad, Hercules, CA, USA), and then analyzed in an ELISA reader (model 3550 UV ELISA reader; Bio-Rad). For proliferation analysis of healthy and transformed keratinocytes upon peptide or chemical inhibitor treatment, 1 × 10^4^ healthy keratinocytes, CAL27 and CAL33 cells were plated in 96-well plates in quadruplicate for each condition. After 1 day, medium was changed with fresh medium in the presence or not of Ctrl1 or KIR-ESS peptides (40 μM final concentration) or 10 μM STAT3 (S3I-201, Santa Cruz) or 20 μM Erk1/2 (PD98059, Calbiochem). After 1-h pre-incubation, cells were treated with IL-22 or left unstimulated, cultured for other 24, 48 and 72 h, and the number of viable cells determined by CyQUANT Cell Proliferation Assay, accordingly to manufacture's protocol. Fluorescence intensities were detected by using a VICTOR3 Multilabel Plate Reader (PerkinElmer, Waltham, MA, USA).

### Scratch assay

Cultured keratinocytes and SCC cell lines were grown at 100% confluence on type IV collagen (20 μg/ml), and then incubated with mitomycin C (5 mg/ml) (Sigma-Aldrich) for 1 h. Cell monolayers were scratched with the tip of a p-200 pipette to create uniform cell-free zones, and, then, pre-treated with appropriate concentrations of SOCS3 mimetic or control peptides, or with 10 μM STAT3 (S3I-201, Santa Cruz) and 20 μM Erk1/2 (PD98059, Calbiochem) inhibitors for 1 h, before treating by 50 ng/ml of IL-22. Microscopy pictures were taken with a digital camera at different time points following IL-22 treatment. The residual gap between migrating keratinocytes was measured with a computer-assisted image analysis system (Axiovision; Zeiss, Oberkochen, Germany), and expressed as percentage of the initial scratched area.

### Tumor growth in nude mice

Eight-week-old male CD-1 nude mice (Charles River Laboratories, Calco, Italy) were injected subcutaneously with 5 × 10^6^ CAL27 cells and tumors were allowed to grow for four-five days. Once tumors were established, mice were then randomized in six groups (five mice for each group) and treated or not with 350 ng IL-22 or 1 × PBS once a day for 7 days, in presence of peptide vehicle (PBS), Ctrl1 or KIRESS peptides (100 mg), by injecting the compounds on the tumor boundaries. Tumor dimensions were measured every day and their volumes were calculated from caliper measurements of two orthogonal diameters (x and y, larger and smaller diameters, respectively) by using the formula volume = xy^2^/2 and normalized to the volume at first day of treatment. The whole experiment was repeated twice with similar results. All mouse procedures were carried out in accordance with institutional standard guidelines. The experimental design has been authorized by the Italian Health Minister (protocol N. 226/2013). Sections were counterstained with Mayer's H&E or subjected to IHC analysis for pY-STAT3 by using a rabbit polyclonal Ab (Cell Signaling Technologies) (1:100). Figures depict one experiment that is representative of all the mice investigated. Slides were analyzed blind by two observers and positive cells were counted in 10 adjacent fields at a magnification of 200 ×.

### Densitometry and statistical analysis

Immunoblots were subjected to densitometry using an Imaging Densitometer model GS-670 (Bio-Rad) supported by the Molecular Analyst software, and band intensities were evaluated in three independent experiments. Data are expressed as densitometric intensity (D.I.) ± SD. Statistical significance was evaluated using Wilcoxon's signed rank test (GraphPad prism Software, La Jolla, CA, USA). Student's *t*-test was used in *in vivo* experiments to compare control- and KIR-ESS- treated groups in presence or not of IL-22, as indicated. Values of *p* < 0.05 were considered significant for all experiments.
